# Use of Liposomal Bupivacaine in Pediatric Peripheral Nerve Blocks after Traumatic Amputation

**DOI:** 10.1155/2021/8092204

**Published:** 2021-08-16

**Authors:** Gregory M. Halenda, Stylianos Voulgarelis

**Affiliations:** Department of Anesthesiology, Division of Pediatric Anesthesiology, Children's of Wisconsin, Medical College of Wisconsin, Milwaukee, Wisconsin, USA

## Abstract

Liposomal bupivacaine has been explored for indications in regional anesthesia, but little has been reported about its use in pediatric patients. In March 2021, the FDA approved an indication for liposomal bupivacaine as an infiltrated local anesthetic in children older than the age of six. Despite this recently expanded indication, the literature lacks reports of use for peripheral nerve blockade in children. We describe a case where liposomal bupivacaine was used for femoral and sciatic nerve blocks in a 5-year-old child with traumatic amputation of his lower leg. Pain control was excellent, with no pain or opioid use reported during the first 62 hours. After the regional anesthesia subsided, the patient required in total 4 oral doses of oxycodone 0.1 mg/kg prior to discharge. The patient did not develop chronic pain or phantom limb syndrome. While liposomal bupivacaine is not currently FDA-approved for peripheral nerve blockade in children, this case highlights a potentially effective use of this drug and possible area for further investigation.

## 1. Introduction

Liposomal bupivacaine (EXPAREL, Pacira Pharmaceuticals) is a newer drug formulation that prolongs the action of traditional bupivacaine. Previously FDA approved for adult populations, and only for tissue infiltration or interscalene nerve blocks, the drug also recently gained FDA approval in pediatric patients older than six years of age in March 2021. Nonetheless, many centers are expanding use in further off-label indications. Scant literature exists regarding liposomal bupivacaine for adult peripheral nerve blockade, or pediatric tissue infiltration, and none in pediatric peripheral nerve blockade, to the authors' knowledge. We present a case of peripheral nerve blockade with liposomal bupivacaine that successfully controlled post-amputation pain in a 5-year-old pediatric patient and provided at least 62 hours of post-op analgesia. The patient's parents provided written consent for publication.

## 2. Case Description

A 5-year-old, 25 kg boy presented to a level-1 pediatric trauma center after partial amputation injury to his lower leg during a lawnmower accident. On arrival, the patient was observed to have pallor, tachycardia, and hypotension. Massive transfusion was initiated, and the patient rapidly received resuscitation with 3 units of PRBC, 2 units of FFP, and 1 pack of platelets. The patient was transferred directly from the trauma bay to the operating room for control of hemorrhage and completion of amputation. A below-knee amputation was performed. Intraoperative pain mitigation strategies included fentanyl 200 mcg, magnesium 1 gm, and ketamine 50 mg. Because of the need for massive transfusion and potential challenges with pain and sedation, the patient was admitted to the PICU. Through POD0, the patient remained intubated and mechanically ventilated, with the ICU sedation strategy consisting of ketamine infusion supplemented by intermittent doses of lorazepam.

Early on POD1, placement of peripheral nerve blockade to minimize acute and chronic pain was considered. This was not feasible immediately intra- or post-op due to the emergent nature of the procedure, potential coagulopathy, and lack of consent. After discussion with the parents and the surgeon, and prior to extubation, ultrasound-guided peripheral nerve blocks of the femoral and sciatic nerves were performed. Liposomal bupivacaine was used in both nerve blocks, with a total of 8 ml of 1.33% liposomal bupivacaine diluted in 8 ml of preservative-free normal saline (approximately 4 mg/kg bupivacaine). Drug mixture was injected approximately evenly between both femoral and sciatic sites. After administration of regional anesthesia, sedation was halted, and the patient was extubated. The total duration of post-op ventilation was 15 hours. The patient was also started on doses of oral acetaminophen (15 mg/kg q6h), gabapentin (100 mg q8h), and ibuprofen (10 mg/kg q6h).

Complete control of pain was noted, with pain scores of 0 for every observation, until the first instance of pain at 62 hours after nerve block administration. Pain scores were reported in the range of 0–10 on the FLACC (Face, Legs, Activity, Cry, Consolability) scale every hour while awake. While evaluation was somewhat obscured by the patient's developmental status and cooperation, subjective and objective evaluations indicated that the femoral distribution of blockade had started to recede around that time, although sciatic distribution appeared still in effect past 62 hours. No signs or symptoms of toxicity were observed at any point. Total opioid dosage from extubation to discharge from the hospital consisted of 4 oral doses of oxycodone (0.1 mg/kg/dose). Pain remained under excellent control throughout hospitalization. After discharge, follow-up at 1-week and 1-month periods was significant for absent pain or phantom limb symptoms. Additionally, no residual weakness, sensory deficit, or other complications have been noted.

## 3. Discussion

Amputation is associated with significant risks for acute and chronic pain. While no single regimen may always prevent post-amputation pain and phantom limb pain, the topic remains an area of focus [1]. Regional anesthesia is a strategy to prevent and manage pain. Peripheral nerve blocks, when appropriate, have elegant advantages including specificity of effect, relative lack of invasiveness, and hemodynamic stability, as compared to neuraxial anesthesia. Using continuous infusion catheters for peripheral nerve blockade include challenges with placement, dislodgement, infection, and burden of follow-up. For these reasons, “single-shot” peripheral nerve blocks may be favored, although duration of action is necessarily limited.

Liposomal bupivacaine has drawn considerable interest with the aim of bridging a gap between single-shot and continuous nerve blocks, although the literature on this topic is still maturing. In terms of effectiveness for pain control, a Cochrane review on the topic declared insufficient evidence to compare liposomal versus plain bupivacaine for peripheral nerve blocks [2]. More recently, a 2021 meta-analysis has found liposomal bupivacaine not superior to plain bupivacaine for peripheral nerve blocks [3]. Even more sparse are data in pediatric patients, although narrow indications such as infiltration in spine fusion are being explored [4]. While detailed discussion of the recent meta-analysis [3] is beyond the scope of this report, it remains possible that liposomal bupivacaine may be useful in certain clinical situations. For example, in particularly active children who are not developmentally mature, peripheral nerve catheters may add significant burden of entanglement with extra dressings and infusion pumps. Not only does the presence of an infusion pump necessarily increase the number of attached devices, but also the catheter itself is prone to dislodgement from the intended site of action. For these reasons, the authors prefer single-shot techniques for peripheral nerve blockade when feasible in the pediatric population. While one could extrapolate the adult data to assume that liposomal bupivacaine is not superior to bupivacaine in the pediatric population, this has not yet been proven and could be an area for further investigation.

In terms of safety, our patient did not experience any side effects or adverse events related to regional anesthesia. Existing data in children suggest that liposomal bupivacaine is safe with no LAST events reported [5] Interestingly, as reported by Cohen et al., when the criteria were reduced to a single symptom suggestive of LAST rather than at least two, liposomal bupivacaine had a relative risk of 2.4 compared to bupivacaine. Still, given the small numbers of potential complications and the relatively vague symptoms included such as dizzyness or drowsiness, it is difficult to draw overarching conclusions. Adult pharmacokinetic data may suggest that the liposomal formulation could convey less toxicity than the standard formulation due to a lower peak release [6]. Our institution, a tertiary children's hospital, has explored a variety of uses, including infiltration, plane blocks (erector spinae [7], serratus anterior, transversus abdominis, and quadratus lumborum), and adductor canal blocks in teenage children, with no significant complications reported (unpublished data).

## 4. Conclusion

In this case, to the authors' knowledge, we present the first usage of liposomal bupivacaine for major peripheral nerve blockade in a pediatric patient. While the efficacy of liposomal bupivacaine compared to bupivacaine is a controversial topic, this patient experienced longer control of pain than would have been typically feasible with nonliposomal bupivacaine. Liposomal bupivacaine administered through femoral and sciatic peripheral nerve blocks provided at least 62 hours of effective analgesia in this patient undergoing below-knee amputation. No adverse events or side effects were noted. Minimal opioids were required after resolution of regional anesthesia. In a patient population where single-shot nerve blockade is desirable, liposomal bupivacaine may be a reasonable choice. More data for use in pediatric patients would be helpful in clarifying the drug's role in our practice.

## Data Availability

The results of the statistical analysis of the data used to support the findings of this study are included within the article. The raw data including FLACC and RASS scores as well as the analytical methods used may be deidentified for patient name and released upon request to the corresponding author.

## Abbreviations

FDA: Food and Drug Administration
PRBC: Packed red blood cells
FFP: Fresh frozen plasma
POD: Postoperative day
PICU: Pediatric intensive care unit.

## References

[1] Ahuja V., Thapa D., Ghai B. (2018). Strategies for prevention of lower limb post-amputation pain: a clinical narrative review. *Journal of Anaesthesiology, Clinical Pharmacology*.

[2] Hamilton T. W., Athanassoglou V., Trivella M. (2016). Liposomal bupivacaine peripheral nerve block for the management of postoperative pain. *Cochrane Database of Systematic Reviews*.

[3] Hussain N., Brull R., Sheehy B. (2021). Perineural liposomal bupivacaine is not superior to nonliposomal bupivacaine for peripheral nerve block Analgesia. *Anesthesiology*.

[4] Chughtai M., Sultan A. A., Hudson B. (2020). Liposomal bupivacaine is both safe and effective in controlling postoperative pain after spinal surgery in children. *Clinical Spine Surgery: A Spine Publication*.

[5] Cohen B., Glosser L., Saab R. (2019). Incidence of adverse events attributable to bupivacaine liposome injectable suspension or plain bupivacaine for postoperative pain in pediatric surgical patients: a retrospective matched cohort analysis. *Pediatric Anesthesia*.

[6] Hu D., Onel E., Singla N., Kramer W. G., Hadzic A. (2013). Pharmacokinetic profile of liposome bupivacaine injection following a single administration at the surgical site. *Clinical Drug Investigation*.

[7] Voulgarelis S., Halenda G. M., Tanem J. M. (2021). A novel use of liposomal bupivacaine in erector spinae plane block for pediatric congenital cardiac surgery. *Case reports in anesthesiology*.

